# Risk factors for diagnosed noma in northwest Nigeria: A case-control study, 2017

**DOI:** 10.1371/journal.pntd.0006631

**Published:** 2018-08-23

**Authors:** Elise Farley, Annick Lenglet, Cono Ariti, Nma M. Jiya, Adeniyi Semiyu Adetunji, Saskia van der Kam, Karla Bil

**Affiliations:** 1 Public Health Department, Médecins Sans Frontières, Operation Centre Amsterdam, Amsterdam, The Netherlands; 2 Department of Public Health Medicine, University of Cape Town, Cape Town, South Africa; 3 Centre for Medical Education, Cardiff University School of Medicine, Cardiff, United Kingdom; 4 Department of Paediatrics, Usmanu Danfodiyo University Teaching Hospital, Sokoto, Nigeria; 5 Department of Plastic Surgery, Usmanu Danfodiyo University Teaching Hospital, Sokoto, Nigeria; 6 Department of Clinical Services, Noma Children’s Hospital, Sokoto, Nigeria; University of California San Diego School of Medicine, UNITED STATES

## Abstract

**Background:**

Noma (cancrum oris), a neglected tropical disease, rapidly disintegrates the hard and soft tissue of the face and leads to severe disfiguration and high mortality. The disease is poorly understood. We aimed to estimate risk factors for diagnosed noma to better guide existing prevention and treatment strategies using a case-control study design.

**Methods:**

Cases were patients admitted between May 2015 and June 2016, who were under 15 years of age at reported onset of the disease. Controls were individuals matched to cases by village, age and sex. Caretakers answered the questionnaires. Risk factors for diagnosed noma were estimated by calculating unadjusted and adjusted odds ratios (ORs) and respective 95% confidence intervals (CI) using conditional logistic regression.

**Findings:**

We included 74 cases and 222 controls (both median age 5 (IQR 3, 15)). Five cases (6.5%) and 36 (16.2%) controls had a vaccination card (p = 0.03). Vaccination coverage for polio and measles was below 7% in both groups. The two main reported water sources were a bore hole in the village (cases n = 27, 35.1%; controls n = 63, 28.4%; p = 0.08), and a well in the compound (cases n = 24, 31.2%; controls n = 102, 45.9%; p = 0.08). The adjusted analysis identified potential risk and protective factors for diagnosed noma which need further exploration. These include the potential risk factor of the child being fed pap every day (OR 9.8; CI 1.5, 62.7); and potential protective factors including the mother being the primary caretaker (OR 0.08; CI 0.01, 0.5); the caretaker being married (OR 0.006; CI 0.0006, 0.5) and colostrum being given to the baby (OR 0.4; CI 0.09, 2.09).

**Interpretation:**

This study suggests that social conditions and infant feeding practices are potentially associated with being a diagnosed noma case in northwest Nigeria; these findings warrant further investigation into these factors.

## Author summary

Noma or cancrum oris is an orofacial gangrene that rapidly disintegrates the hard and soft tissue of the face. Little is known about noma as most cases live in underserved, difficult to reach locations. There is a dearth of literature on the risk factors for the development of noma. Médecins Sans Frontières (MSF) in collaboration with the Nigerian Ministry of Health runs projects at the Noma Children’s Hospital in Sokoto. A case control study was conducted in northwest Nigeria to explore exposures associated with diagnosed noma using unadjusted and adjusted conditional logistic regression models. Potential risk and protective factors for diagnosed noma were identified and these findings need further exploration. The study identified that feeding pap to the child every day was a potential risk factor for diagnosed noma (possibly a proxy for poor variety in the diet). The following potential protective factors for diagnosed noma were identified: the mother being the primary caretaker, the caretaker being married, and colostrum being given to the baby. Noma is a neglected disease, and current risk factors suggest that intervention efforts could be more effective by focussing on access to health care, the benefits of breastfeeding and a varied diet. However, more research is needed in order to better understand the pathogenesis of this disease in order to improve prevention, early detection and treatment.

## Introduction

Noma or cancrum oris, is a poorly understood, rapidly progressing gangrenous infection of the oral cavity, associated with a high mortality rate [1]. It mostly affects children under the age of five years [2]. It is estimated that up to 90% of noma cases die [3], and those who survive have severe facial disfigurements [2]. These can result in multiple physical impairments such as difficulty speaking, swallowing, eating, seeing and breathing which can lead to stigmatization in their communities [4]. Noma is thought to be most prevalent along the noma belt which stretches from Senegal to Ethiopia [4], however noma cases have recently been reported in the United Kingdom [5], United States [6], Afghanistan [7], South Korea [8] and Laos [9]. A northwest Nigerian based study concluded that the incidence of noma is estimated to be 6.4 per 1000 children [10], and the World Health Organization (WHO) estimates that 140 000 children contract noma each year globally [2].

Little is understood about noma as most cases live in underserved, difficult to reach locations, many cases go undiagnosed and the mortality rate is so high. Previous observational studies have suggested that risk factors for development of noma include malnutrition, low birthweight, absence of breastfeeding, poor oral hygiene, co-morbidities, proximity of livestock to area of residence, large family size, access to unsafe drinking water and living in a village with a high prevalence of acute necrotising gingivitis [4,11–13]. Recently, an increased incidence of noma has been reported in higher resource settings in patients with immunosuppressive diseases such as human immunodeficiency virus (HIV) [5,6,8,14,15].

Since 2015, Médecins Sans Frontières (MSF) has collaborated with the Nigerian Ministry of Health (MoH) to treat noma patients identified across the northwest of Nigeria, in Sokoto. The programme provides nutritional, psychosocial (for the patient and their families) and medical care to prepare noma patients for the required surgical interventions at the Noma Children’s Hospital. These are conducted by MoH and MSF surgical teams on a routine basis; since August 2015, the programme has treated 227 noma patients. We conducted a case-control study to identify risk factors for diagnosed noma in terms of demographic characteristics, medical history, socio-economic-behavioural aspects and access to health care in order to better guide existing prevention and treatment strategies for this neglected disease.

## Methods

### Study location

The study was conducted in Sokoto and Kebbi states, which are located in northwest Nigeria.

### Study population

Cases were defined as patients with diagnosed noma admitted to the Noma Children’s Hospital between May 2015 and June 2016 who were under 15 years of age at self-reported onset of the disease. Controls were individuals matched to cases by village of residence, current age (+/- 2 years) and sex.

### Sample size

Our aim was to include all cases enrolled at the Noma Children’s Hospital in the year before data collection, and we calculated that with a sample size of 67 cases and 200 controls (three controls per case), we would be able to estimate an odds ratio (OR) of 2.5 for suspected risk factors with a power of 80% and 60% of controls being exposed to that risk factor. Controls were selected from houses neighbouring those in which the cases and their families live.

### Data collection

Seventy-eight percent of cases and controls were younger than 18 years of age, their parents or caretakers were asked to participate in the interviews using a structured questionnaire. The questionnaire covered the sociodemographic characteristics of the cases and controls (age, gender, education, employment, total household members), their current living conditions (water source, proximity to livestock, material of houses) and their vaccination history (read on vaccination card if available). Additionally, parents and/or caretakers were asked to respond to questions pertaining to the duration of breastfeeding after the cases and controls were born and other nutrition-related practices during the neonatal period and current practices. The health status, access to health care and healthcare seeking behaviour for the case or control in the previous 12 months were also assessed. Finally, all cases and controls aged less than five years at the time of interview had a mid-upper arm circumference (MUAC) measurement taken at the time of the interview.

The questionnaire was formatted in Kobo Collect (http://www.kobotoolbox.org/) and uploaded to tablets for mobile data collection purposes. Completed questionnaires were uploaded daily to a secure MSF server through an internet connection. The study coordinator verified all completed questionnaires on a daily basis for data consistency and quality.

### Data analysis

We calculated the frequencies and respective proportions for all categorical variables and used chi-square tests for comparison of these variables between cases and controls. For continuous variables, we calculated means with standard deviation or medians and interquartile ranges (depending if approximately Normally distributed) for cases and controls separately, and used t-tests to compare Normally distributed variables, and Kruskal Wallis tests for non-Normally distributed variables.

Food variables for current feeding practices were categorised as animal products (meat, milk, egg), grains (fura, mashed rice, millet, corn, bread) and vegetables (sweet potato, beans, bean cake, moringa leaf with ground nut cake, cassava). Respondents could answer with ‘Yes’, ‘No’ and ‘Don’t know’. We grouped all responses for ‘No’ and ‘Don’t know’ into a single category. To investigate the impact of this grouping, we conducted a sensitivity analysis for each of these variables. As the results showed that the direction of association remained the same, we retained this grouping as the reference category.

We estimated risk factors for being a diagnosed noma patient by comparing odds of exposure in cases and controls using unadjusted and adjusted conditional logistic regression to calculate Odds Ratios (ORs) and their respective 95% confidence intervals (CI) and p-values. The adjusted conditional logistic regression model was constructed using all risk factors that had a p-value of <0.2 in the unadjusted analysis and sufficient outcomes in each category. Variables were eliminated from the adjusted model using a manual backwards stepwise approach [16], and adjusted models were compared using the likelihood ratio test, any variable with a p-value under 0.2 was kept in the model. All data analyses were conducted with Stata 14 (StataCorp, College Station, TX, USA).

### Ethics

The MSF Ethical Review Board approved the study protocol (study 1710), as did the Usmanu Danfodiyo University Teaching Hospital (UDUTH) Health Research and Ethics Committee in Nigeria (UDUTH/HREC/2017/No.595) and the Ministry of Health in both Sokoto (SKHREC/032/017) and Kebbi (MOH/SUB/4027/Vol.I/14) states. All interviewees were over the age of 18 and written informed consent was provided by each participant (for participants who were illiterate, the consent form was read aloud to them and a thumb print was then requested). All participants were assured that there was limited risk of harm from participation in this study, and that they were free to withdraw at any point.

## Results

### General findings

Out of the 112 noma patients who had sought care in the programme between May 2015 and June 2016, we identified 87 who lived in Kebbi and Sokoto states and were eligible for inclusion in the study. Of these, 10 could not be located, and we managed to interview 77 cases (88.5%). We were unable to reach the village of three identified cases for logistical reasons. Thus, the final analysis included 74 noma cases and 222 controls. Six of the cases had passed away in the time between discharge and the interview; the interviews were still conducted with their caretakers. At the time of first admission to the hospital, 17 of these cases had acute noma, 57 had inactive noma, two had trismus and one had no diagnosis noted at time of admission to the hospital. Twenty one cases were hospitalized at the time of interview and the remaining 56 were interviewed in their home villages.

As expected, there were no significant differences in matching variables (sex and age of child) between cases and controls. The control group differed from the case group in that caretakers were younger, their family sizes were smaller (and houses in the compound fewer) and most of their houses were made of mud (which might be a proxy for higher socio-economic status) (Table 1). Also, the respondents for the control group were more frequently the mother of the child (Table 1). The self-reported median age of onset of noma amongst cases was 2.0 (IQR 2.0, 3.0).

**Table 1:** Sociodemographic characteristics of cases and controls (p-values from chi-squared, t-test or kwallis).

		*Controls (N = 222)*	*Cases (N = 77)*	*P-value*
Child age at time of interview, Median (IQR)		5.0 (3.0, 15.0)	5.0 (3.0, 15.0)	0.94
Caretaker sex (female)		113 (50.9%)	37 (48.1%)	0.67
Caretaker age	18–25	147 (68.7%)	37 (48.7%)	0.002
	35–90	67 (31.3%)	39 (51.3%)	
	Missing	8	1	
Child first born		33 (14.9%)	13 (16.9%)	0.67
Family size, Mean (SD)		11.5 (8.8)	15.8 (13.8)	0.0016
Number of wives, Mean (SD)		2.0 (7.6)	1.6 (0.7)	0.64
Main caretaker	Mother	132 (59.5%)	25 (32.5%)	<0.001
	Other	36 (16.2%)	28 (36.4%)	
	NA	54 (24.3%)	24 (31.2%)	
Caretaker employed	No	76 (45.2%)	33 (51.6%)	0.39
	Yes	92 (54.8%)	31 (48.4%)	
	Missing/ NA	54	13	
Caretaker education	None	66 (29.7%)	28 (43.8%)	0.34
	Educated	102 (46.0%)	36 (56.3%)	
Number of houses in compound, Mean (SD)		3.5 (2.8)	4.5 (3.5)	0.008
Main materials of house walls	Wood, mud, bamboo	175 (78.8%)	41 (53.3%)	<0.001
	Stone, brick, cement	45 (20.3%)	12 (15.6%)	
	Other	2 (0.9%)	24 (31.2%)	

Cases between 6 months and 5 years old had lower mean MUAC measurements (mean 134; SD 20) than controls (mean 142; SD 11; p = 0.002. Six percent of cases (n = 5) had a vaccination card available at the time of interview, compared with 16% (n = 36; p = 0.03) of controls. The vaccination status (based on the vaccination card was) for polio (cases n = 3, 3.9%; controls n = 13, 5.9%; p = 0.07) and measles (cases n = 2, 2.6%; controls n = 15, 6.8%; p = 0.1) was very low for both cases and controls. Parents and caretakers reported that most cases (n = 63, 81.8%) and controls (n = 158, 71.2%; p = 0.22) had been breastfed between one to two years after birth. With respect to the main source of drinking water, parents reported that the two main water sources were a bore hole in the village (cases n = 27, 35.1%; controls n = 63, 28.4%; p = 0.08) and a well in the family compound (cases n = 24, 31.2%; controls n = 102, 46.0%; p = 0.08). In terms of animal ownership, the proportion of case and control families reporting owning donkeys, dogs, sheep, goats and chickens were similar. Case families, however, were less likely to have cows in their compound compared with controls (cases n = 36, 46.8%; controls n = 128, 57.7%; p = 0.09).

### Risk factor analysis

The unadjusted analysis suggested that the likelihood of being a diagnosed noma case increased when the household was large (>10 people), the child was heavy at birth (self-reported weight), breastfeeding occurred for >1 year, first solid food was given after the age of 12 months, the child ate pap (type of porridge staple made from maize, sorghum, or millet), and grains each day. Protective factors against acquiring noma included: the mother being the primary caretaker, the caretaker being married, having a well in the compound, colostrum being given to the child, child first given water over the age of 7 months, all children in the household being alive and the child having taken medication (traditional or biomedicine) in the year preceding the interview (Table 2).

**Table 2:** Unadjusted and adjusted risk factors for being a noma case from conditional logistic regression, Sokoto and Kebbi States, Nigeria 2017.

		Controls (N = 222)	Cases (N = 77)	Unadjusted			Adjusted		
		n (%)	n (%)	OR	95% CI	P-Value	OR	95% CI	P-Value
Primary Caretaker is mother		132 (60%)	25 (32%)	0.2	0.1, 0.5	<0.001	0.08	0.01, 0.5	0.007
Caretaker married		158 (71%)	14 (18%)	0.03	0.01, 0.09	<0.001	0.006	0.0006, 0.5	<0.001
Child first born		33 (15%)	13 (17%)	1.2	0.6, 2.5	0.6			
Caretaker Educated		102 (46%)	36 (47%)	1	0.6, 1.8	1			
Caretaker Employed		92 (41%)	31 (40%)	0.9	0.5, 1.7	0.9			
Total household members	1–9	113 (51%)	29 (38%)	Reference					
	10 or above	109 (49%)	48 (62%)	1.8	1.006, 3.09	0.05	2.06	0.8, 5.3	0.1
COMPOUND LIFE									
Water Source	Other	120 (54%)	53 (69%)	Reference					
	Well in compound	102 (46%)	24 (31%)	0.5	0.2, 0.9	0.04			
Water treated		73 (33%)	24 (31%)	0.9	0.5, 1.6	0.8			
Electricity	Yes	77 (35%)	25 (32%)	0.5	0.2, 1.4	0.2	0.2	0.04, 1.5	0.1
Livestock in compound	1–3	100 (45%)	40 (52%)	Reference					
4 or more animal species		122 (55%)	37 (48%)	0.8	0.4, 1.4	0.4			
NUTRITION									
Colostrum	No	19 (9%)	19 (25%)	Reference			Reference		
	Yes	149 (67%)	44 (57%)	0.3	0.2, 0.7	0.002	0.4	0.09, 2.09	0.07
	Don’t know	54 (24%)	14 (18%)	0.05	0.005, 0.4		0.001	3.3e-06, 0.5	
Breastfed length	7–12 mnth, other	64 (29%)	14 (18%)	Reference					
>1 year and <2 years		158 (71%)	63 (82%)	16.9	2.1, 134.5	0.008			
Age child first given water	0–6	126 (57%)	53 (69%)	Reference					
	7 or above mnths	26 (12%)	6 (8%)	0.4	0.2, 1.2	0.03			
	Don’t know	70 (32%)	18 (23%)	0.2	0.07, 0.8				
Age first given solid food	0–11	112 (51%)	44 (57%)	Reference			Reference		
	12 mnths or after	38 (17%)	16 (21%)	1.2	0.6, 2.3	0.03	5.07	0.9, 26.08	0.07
	Don’t know	72 (33%)	17 (22%)	0.2	0.05, 0.7		0.2	0.01, 2.9	
Pap every day (Y)		124 (56%)	55 (71%)	3.5	1.5, 8.2	0.004	9.8	1.5, 62.7	0.02
Food eaten at least once a day (Y) (ref = no/ don’t know)									
Animal products		28 (13%)	10 (13%)	0.9	0.4, 2.1	0.9			
Grains		165 (74%)	62 (81%)	5.9	1.2, 29.7	0.03			
Vegetable		57 (26%)	21 (27%)	1	0.5, 1.9	1			
Drinks		94 (42%)	36 (47%)	1.2	0.7, 2.2	0.6			
Child, family eat same meals (Y)		165 (74%)	61 (79%)	3.4	0.8, 13.6	0.09			
HEALTH									
Births in total	0–5	99 (46%)	30 (39%)	Reference					
	6 or more	69 (31%)	34 (44%)	1.4	0.8, 2.6	0.5			
	Don’t know	54 (24%)	13 (17%)	5.80E-08	0, -				
Births Alive	Some Died	64 (29%)	43 (56%)	Reference					
	All still alive	103 (46%)	20 (26%)	0.4	0.2, 0.6	0.003			
	Don’t know	55 (25%)	14 (18%)	3.90E-08	0, -				
Birthweight	Light	27 (12%)	9 (12%)	Reference					
	Heavy	78 (35%)	39 (51%)	1.6	0.7, 3.7	0.03			
	Don’t know	117 (53%)	29 (38%)	0.7	0.3, 1.7				
Meds in previous year(Y)		127 (57%)	32 (42%)	0.4	0.2, 0.7	0.006	0.3	0.07, 1.6	0.2

Due to collinearity, the following variables were not included in the adjusted analysis: the food group variable “Grains” (fura, mashed rice, millet, corn, bread), material the walls of the house was made out of, wealth score, measles, polio, one or more vaccination being reported on a vaccination card. In the adjusted analysis, eating pap every day remained strongly associated with being a risk factor for diagnosed noma (OR 9.8; CI 1.5, 62.7; p = 0.02) as did a later age of first solid food (>12 months) (OR 5.07; CI 0.9, 26.08; p = 0.07). Variables that were protective against being a case included the mother being the primary caretaker (OR 0.08; CI 0.01, 0.5; p = 0.007), the caretaker being married (OR 0.006; CI 0.0006, 0.5; p = <0.001) and colostrum being given to the baby (OR 0.4; CI 0.09, 2.09; p = 0.07) (Table 2).

## Discussion

This study has highlighted potential risk and protective factors associated with being a diagnosed noma case in northwest Nigeria, including caretaker demographics and infant feeding practices. This identification aims to shed light on potential useful areas for further investigation. Similar risk factors have been suggested including the absence of breastfeeding [12], dietary habits [17], unsafe drinking water, limited access to high quality health care, high infant mortality [4], and food security [11]. A 2017 review noted that measles vaccination was a protective factor for noma [11], but we were unable to corroborate this as the estimated vaccination coverage in both cases and controls was too low for a relevant comparison to be conducted. Our adjusted analysis did not identify an association between noma and household size or water source as reported elsewhere [9,12,18], however, the unadjusted analysis suggested a possible association between these exposures and diagnosed noma and therefore warrants further investigation. We were also unable to confirm other reported risk factors for noma including high numbers of previous pregnancies in the mother, [12] pre-existing illness, malnutrition [17], poor oral hygiene practices [1,2,4,9–11,18–23], proximity of livestock to the area of residence and low birthweight [2,4,7,12,18,20,23,24], these require further exploration.

One novel observation of the study was some weak evidence for the possible protective nature of colostrum. There are inherent health benefits associated with colostrum as it is rich in antibodies that confer passive immunity and growth factors which have been shown in recent studies to assist in the treatment of autoimmune disorders and gastrointestinal conditions [25]. Recent case reports have highlighted the global problem of noma and in some cases a relation to concomitant infections including HIV-positive patients in London [5] and in the United States [6], and a Korean child with Crohn’s disease [8]. These reports offer insight into the disease and strongly suggest that immunosuppression plays a role in the causal pathway of noma development which supports the finding of the protective nature of colostrum. However, it should be noted that these are clinical case reports and not analytical epidemiological studies. It is also possible that mothers who give their children colostrum are also more likely to follow other health messaging. Both aspects would have a favourable impact on the overall health status of an infant and therefore render the child to be at a lower risk of noma development. Our findings on this point were not definitive but this could be a useful point for future research.

The finding in the unadjusted analysis that having taken medication in the year prior to the study was a protective factor could indicate that having access to health care plays a role in noma development. This could be due to health care proximity or possibly due to improved socio economic status which would allow the families to pay for transport to get to health care and/or healthcare provision. Those who develop noma would possibly have fewer possibilities to seek care for other diseases or the beginning stages of noma, and as such are at higher risk of developing the disease.

The finding that eating pap every day is a risk factor for diagnosed noma development, could be due to the fact that pap is the food usually used for weaning in Nigeria, it has a low nutrient density and has been reported to be at risk of unhygienic handling [26]. Two microbiological analyses of foods commonly used in weaning (including pap) in Nigeria, showed bacterial contamination at higher levels than international guidelines recommend [27,28]. All of these factors mean that during the weaning period, the use of pap can predispose infants to infections and subsequently high mortality rates [26]. Possible solutions to reduce this exposure, would be to strengthen education about hygienic food preparation [27], the benefits of alternate practices such as fermentation which enhances nutritive value of the food [26] or encouraging variety in the diet. Improving feeding during the weaning time is crucial to decrease child malnutrition, mortality rates and to enhance development [28].

The findings showed that in both the case and control groups there was a high proportion of child morbidity and that families where the majority of infants born to the same mother survived, were less likely to have a noma case. This finding might strengthen the role that socio-demographic characteristics of families and households play in the disease dynamics of diagnosed noma. If more children die in households where noma cases are detected, it could indicate that children in these households are also more exposed to unsafe water, poorer standards of living and reduced access to care, as all of these exposures are known to be associated with higher infant mortality [29–34].

The proportion of vaccination coverage in the study population is below the standards recommended by the Nigerian Ministry of Health [35] and WHO [36]. There is evidence that the occurrence of vaccine preventable diseases and malnutrition precede the onset of noma [4,6,9,18–20]. The low coverage of vaccination not only increases the risk of morbidity and mortality from vaccine preventable diseases, but is a contributing factor in immunosuppression which is thought to play a key role in the sequence of events for noma development.

Our study is unique because available evidence around noma manifestation and risk factors for the development of the disease is based on a handful of primary studies [13,12,37], case reports [5,7–9] and reviews [2,4,19]. These studies were methodologically different from ours which could offer reasons for the differing findings. A case control study conducted in Niger came closest to the current study design, although this study had an intake period of 6 years and the controls were not matched on sex [12]. Our results also differ from those of a further Niger-based study [13], which included four villages in the study population and focused on the link between noma and acute necrotising gingivitis. In comparison, our study was conducted over a relatively short time period and was retrospective in nature. Furthermore, our cases and controls were matched on sex in addition to village of residence and age, which might have eliminated some bias that could have been present in the previously mentioned studies. Finally, our study included diagnosed noma cases who were resident across 80 villages in two states of northwest Nigeria, so they represent a much wider geographical area than the previous studies, which might have contributed to the differences in risk factors identified [13].

We attempted to interview all patients cared for at the hospital in the year before data collection, however this was not possible due to several constraints. Even though we managed to exceed our minimum sample size, we identified strong associations between only a minority of the explored risk factors and diagnosed noma. It is likely that the risks associated with individual exposures for noma are weaker than we assumed in the original sample size calculation.

A further limitation is that our risk factor analysis only represents those cases who sought care at the Noma Children’s Hospital in Sokoto. Due to the reported 90% mortality rate [3] and rapid progression of noma, this likely means that the results of the current study are only applicable to the subset of noma cases who experienced the least severe complications from the disease.

Finally, we included all cases who were under the age of 15 at the time of self-reported disease onset. Therefore some cases (and thus controls) were under 15 at the age of onset but were currently > = 18 (13 cases) therefore leading us to interview them personally and not their caretaker. This might have introduced some bias into the study as certain questions around infant feeding practices etc. would only have been possible to be answered by their caretakers, thus leading to missing information on these questions. We tried to mitigate this by conducting sensitivity analyses for these variables which resulted in similar findings.

### Conclusions and recommendations

In conclusion, our case control study suggests that infant and current feeding behaviours as well as caretaker demographics may affect the risk of developing noma. Malnutrition and low vaccination coverage, high morbidity of infectious diseases along with low access to health care are all likely contributing factors.

We recommend that further research is implemented to determine the true burden of noma, and that prospective studies are implemented to better understand the sequence of events contributing to the development of noma. Only with these sets of indicators will it be possible to better formulate and target prevention programmes.

## Supporting information

S1 Checklist STROBE Checklist. (DOCX)
