# Supplementary material for: Risk factors for diagnosed noma in northwest Nigeria: A case-control study, 2017
Source: PLoS Negl Trop Dis. 2018 Aug 23;12(8):e0006631. doi: 10.1371/journal.pntd.0006631 (PMC6107110; doi:10.1371/journal.pntd.0006631)
Supplement: S1 Checklist — (DOCX) [file pntd.0006631.s001.docx]

S1- STROBE Checklist for **Risk factors for diagnosed Noma in northwest Nigeria: A case-control study, 2017**

|  | Item No. | Recommendation | Section | Paragraph |
| --- | --- | --- | --- | --- |
| **Title and abstract** | 1 | (*a*) Indicate the study’s design with a commonly used term in the title or the abstract | Cover Page | Paragraph 1 |
|  |  | (*b*) Provide in the abstract an informative and balanced summary of what was done and what was found | Abstract | Entire Section |
| Introduction | | | |  |
| Background/rationale | 2 | Explain the scientific background and rationale for the investigation being reported | Introduction | Paragraph 1 – 3 |
| Objectives | 3 | State specific objectives, including any prespecified hypotheses | Introduction | Paragraph 3 |
| Methods | | | |  |
| Study design | 4 | Present key elements of study design early in the paper | Introduction | Paragraph 3 |
| Setting | 5 | Describe the setting, locations, and relevant dates, including periods of recruitment, exposure, follow-up, and data collection | Methods | Subsection- Study Location  Paragraph 1 |
| Participants | 6 | (*a*) *Case-control study*—Give the eligibility criteria, and the sources and methods of case ascertainment and control selection. Give the rationale for the choice of cases and controls | Methods | Subsection- Study Population  Paragraph 1 |
|  |  | (*b*) *Case-control study*—For matched studies, give matching criteria and the number of controls per case | Methods | Subsection- Study Population  Paragraph 1 |
| Variables | 7 | Clearly define all outcomes, exposures, predictors, potential confounders, and effect modifiers. Give diagnostic criteria, if applicable | Methods | Subsection- Data Collection  Paragraph 1 |
| Data sources/ measurement | 8* | For each variable of interest, give sources of data and details of methods of assessment (measurement). Describe comparability of assessment methods if there is more than one group | Methods | Subsection- Data Collection  Paragraph 1 |
| Bias | 9 | Describe any efforts to address potential sources of bias | Methods | Subsection- Study Population  Paragraph 1 (discussing matching variables to remove bias) |
| Study size | 10 | Explain how the study size was arrived at | Methods | Subsection- Sample Size  Paragraph 1 |

| Quantitative variables | 11 | Explain how quantitative variables were handled in the analyses. If applicable, describe which groupings were chosen and why | Methods | Subsection- Data Analysis  Paragraph 1 - 3 |
| --- | --- | --- | --- | --- |
| Statistical methods | 12 | (*a*) Describe all statistical methods, including those used to control for confounding | Methods | Subsection- Data Analysis  Paragraph 1 - 3 |
|  |  | (*b*) Describe any methods used to examine subgroups and interactions | Methods | Subsection- Data Analysis  Paragraph 2 |
|  |  | (*c*) Explain how missing data were addressed | Discussion | Paragraph 10 |
|  |  | (*d*) *Case-control study*—If applicable, explain how matching of cases and controls was addressed | Methods | Subsection- Data Analysis  Paragraph 3 |
|  |  | (*e*) Describe any sensitivity analyses | Discussion | Paragraph 10 |
| Results | | | | |
| Participants | 13* | (a) Report numbers of individuals at each stage of study—eg numbers potentially eligible, examined for eligibility, confirmed eligible, included in the study, completing follow-up, and analysed | Results | Subsection- General Findings  Paragraph 1 |
|  |  | (b) Give reasons for non-participation at each stage | Results | Subsection- General Findings  Paragraph 1 |
|  |  | (c) Consider use of a flow diagram | Considered but decided against | |
| Descriptive data | 14* | (a) Give characteristics of study participants (eg demographic, clinical, social) and information on exposures and potential confounders | Results | Subsection- General Findings  Paragraph 2  Table 1 |
|  |  | (b) Indicate number of participants with missing data for each variable of interest | Results | Subsection- General Findings  Table 1 |
| Outcome data | 15* | *Case-control study—*Report numbers in each exposure category, or summary measures of exposure | Results | Subsection- Risk Factor Analysis  Table 2 |
| Main results | 16 | (*a*) Give unadjusted estimates and, if applicable, confounder-adjusted estimates and their precision (eg, 95% confidence interval). Make clear which confounders were adjusted for and why they were included | Results | Subsection- Risk Factor Analysis  Table 2 |
|  |  | (*b*) Report category boundaries when continuous variables were categorized | Results | Subsection- Risk Factor Analysis  Table 2 |
|  |  | (*c*) If relevant, consider translating estimates of relative risk into absolute risk for a meaningful time period | NA | NA |

| Other analyses | 17 | Report other analyses done—eg analyses of subgroups and interactions, and sensitivity analyses | Discussion | Paragraph 10 |
| --- | --- | --- | --- | --- |
| Discussion | | | | |
| Key results | 18 | Summarise key results with reference to study objectives | Discussion | Paragraph 1 |
| Limitations | 19 | Discuss limitations of the study, taking into account sources of potential bias or imprecision. Discuss both direction and magnitude of any potential bias | Discussion | Paragraph 9 - 10 |
| Interpretation | 20 | Give a cautious overall interpretation of results considering objectives, limitations, multiplicity of analyses, results from similar studies, and other relevant evidence | Discussion | Paragraph 1 - 8 |
| Generalisability | 21 | Discuss the generalisability (external validity) of the study results | Discussion | Paragraph 8 |
| Other information | |  | | |
| Funding | 22 | Give the source of funding and the role of the funders for the present study and, if applicable, for the original study on which the present article is based | Funding | Paragraph 1 |

*Give information separately for cases and controls in case-control studies and, if applicable, for exposed and unexposed groups in cohort and cross-sectional studies.

**Note:** An Explanation and Elaboration article discusses each checklist item and gives methodological background and published examples of transparent reporting. The STROBE checklist is best used in conjunction with this article (freely available on the Web sites of PLoS Medicine at http://www.plosmedicine.org/, Annals of Internal Medicine at http://www.annals.org/, and Epidemiology at http://www.epidem.com/). Information on the STROBE Initiative is available at www.strobe-statement.org.
